# Exploring perceptions of what increased gender diversity might bring to the nursing profession

**DOI:** 10.1111/jan.16246

**Published:** 2024-08-16

**Authors:** Julie McMullan, David R. Thompson, Alexx Dixon, Alex Palumbo, Tommy Dickinson, Praise Jourdain, Catherine Monaghan, Barry Quinn

**Affiliations:** ^1^ School of Nursing and Midwifery Queen's University Belfast Belfast UK; ^2^ Florence Nightingale Faculty of Nursing, Midwifery & Palliative Care King's College London London UK

**Keywords:** diversity, gender, interview, nursing, qualitative, research, survey

## Abstract

**Aims:**

To explore university nursing students and academic staff's perceptions of what increased gender diversity might bring to the nursing profession.

**Design:**

An exploratory study with students and academic staff from two university nursing schools.

**Methods:**

Students and staff in two university nursing schools were invited to participate in an anonymous online survey (October–November 2022). The survey focused on gender diversity within nursing and patient care, asking respondents about existing barriers to gender diversity and inviting ideas on increasing gender diversity within nursing. The survey findings helped inform questions, which were used to further explore views and thoughts of gender diversity within nursing through semi‐structured one‐to‐one interviews (January–February 2023).

**Results:**

Nearly, two‐thirds (64%, *n* = 69) of survey respondents strongly agreed/agreed that the lack of gender diversity in nursing negatively impacted delivering care to a gender‐diverse society. Most (84%, *n* = 90) strongly agreed/agreed that increased gender diversity within the nursing workforce would positively contribute to nursing. Three‐quarters (75%, *n* = 74) strongly agreed/agreed that working with a more gender‐diverse nursing workforce would enrich their experience as a nurse. Three themes emerged from the interview data: shared stereotypes; improved care through knowledge, better relations and the presence of a gender‐diverse workforce; a culture of welcome: suggested changes for the future.

**Conclusion:**

Most of those who participated in the study believe there are benefits to be gained from greater gender diversity within nursing. This study provides insight into the lack of gender diversity in the nursing environment, highlights how this might impact patient care and suggests actions to make nursing a more inclusive profession.

**Implications for the Profession and/or Patient Care:**

Practical solutions were suggested, including the greater visibility of trans and non‐binary persons in advertisement and recruitment campaigns to ensure nursing is viewed as a career choice for “all”. Including a space for considered pronouns on university application forms, hospital documentation and name badges. Gender‐neutral titles and uniforms should be considered for all nursing staff, and more gender‐neutral toilets should be made available for staff and patients in university and hospital settings.

**Impact:**

This study addressed the lack of gender diversity in nursing and explored what an increase in gender diversity might bring to the nursing profession. The main findings were: (i) Less than a tenth (9%, *n* = 12) of respondents described their team of colleagues as being “extremely gender diverse”. (ii) Most (84%, *n* = 90) strongly agreed/agreed that increased gender diversity within the nursing workforce would be a positive attribute to nursing. (iii) Three‐quarters 75% (*n* = 74) strongly agreed/agreed that working with a more gender‐diverse nursing workforce would enrich their experience as a nurse. (iv) Many stereotypes still exist in nursing and tackling them is important to make the profession more inclusive. (v) A nurse's gender can impact the care they provide. (vi) A more gender‐diverse workforce would better reflect the population it serves. (vii) This research will have an impact on the nursing profession globally.

**Reporting Method:**

The consolidated criteria for reporting qualitative studies (COREQ) was used as a guide throughout data collection and analysis.

**Patient or Public Contribution:**

Every step of the study, including the survey and interview schedule, was co‐constructed using an iterative approach with service users, students and staff who had personal experience of gender diversity and were a core part of the study team.

**What does this paper contribute to the wider global clinical community?**

- Education and training on gender diversity should be mandatory for all those entering the nursing profession.
- A zero‐tolerance policy for gender discrimination should be implemented within the nursing profession and in all healthcare practices.
- Nursing staff should be encouraged to display their pronouns on a name badge or within their email signature to highlight the presence of gender diversity.

## 1 INTRODUCTION

In many parts of the world, there is a widely held assumption that a person can only be one of two genders. However, today, more people than ever are living their lives without strict attachment to one gender or are transitioning from one gender to another (Monro, 2005). The United Nations Mandate (2022) on “sexual orientation and gender diversity” recognizes the richness of gender diversity within the global society. Gender diversity is a term that recognizes the diversity of gender identities, which include but are much broader than the binary notion of woman or man. It recognizes the freedom of each person to be authentic to be themselves and to recognize and choose their gender identity, the fluidity to explore one's gender and the choice to not conform to being identified by gender at all (UNDP Gender Equality Strategy 2022–2025).

Worldwide, patient populations in healthcare environments are increasing in gender diversity (Armada & Hubbard, 2010). The idea of gender diversity is not new. Clifton et al. (2018) argued that whilst the gender imbalance in nursing has been noted since the mid‐1940s in the United Kingdom (UK) when there were separate registers kept for women and men working as nurses, little has been done to improve the uptake into the profession to represent the rich and diverse gender society in which nursing exists.

Quinn et al. (2022) suggested that now is the time to examine more closely what greater gender diversity can bring to nursing and healthcare and to identify what is missing when that diversity is not present, visible or acknowledged. They suggested that making some simple changes to convey an inclusive and welcoming culture could assist in developing a nursing profession that is attractive to transgender, queergender and non‐binary people and easier for them to participate in, and that potentially represents a safer space in which people with diverse gender identities feel able to share their skills and knowledge. This could make nursing a more inclusive profession.

## 2 BACKGROUND

Nursing practice and education are embedded within broader social and healthcare systems, whose practices have frequently assumed the alignment of gender with birth sex and often fail to adequately recognize the implications that gender identities have for comprehensive and appropriate healthcare delivery (Bauer et al., 2009, 2014; Eliason et al., 2010; Lim & Levitt, 2011). Negative experiences in healthcare create difficulties for those with diverse gender identities to access healthcare (Merryfeather & Bruce, 2014) or cause them to conceal their gender identity from healthcare professionals (Bachman & Gooch, 2018), contributing to their invisibility in healthcare. More awareness is needed of the requirements of this diverse population group (Carabez et al., 2016; Lim et al., 2015; Merryfeather & Bruce, 2014).

As society continues to acknowledge and explore the richness of gender diversity, nursing, as a profession, has an opportunity to actively engage in this important aspect of what it means to be human and one's identity. This includes recognizing the emotional, social, cultural, behavioural and spiritual aspects of each person's gender identity.

The lack of gender diversity in nursing has long been a cause for concern (Yip et al., 2021). Despite much discussion and promotional activity, contemporary nursing is and continues to be perceived as a profession largely served by women (Kirlew et al., 2020). Consequently, the lack of gender diversity in nursing may contribute to a lack of awareness of how to respond to the individual person and the presence of diverse gender identities. This could also be partly due to the lack of attention given to this diverse population in undergraduate and postgraduate nursing and medicine education (Lim et al., 2015; Parameshwaran et al., 2017). A more visibly gender‐diverse workforce might not only enrich nursing as a profession and improve how it is perceived but also help people feel more accepted and understood, enabling them to seek support for their health and social care needs (McCann & Monaghan, 2020).

In some nursing and medical schools, along with other healthcare providers, nurses and doctors are gaining knowledge of this reality's physiological and psychological implications. However, other current understanding of the scope of gender diversity and the personal impact on individuals who may feel they are not recognized. Their unmet needs are still limited, and confusion often results around what vocabulary to use (Alegria, 2011; Bradley‐Springer, 2009; Eliason et al., 2010; Fish, 2010). Without meaning to, nurses may cause unnecessary and unintended harm when providing care for people who do not fit within the categories of “man” and “woman” due to a lack of understanding and experience. If nurses are not educated about gender diversity and do not experience this within their workforce, they may face difficulties when caring for people who have come to recognize that their gender designated at birth was incorrect or people who may express their gender differently from the culturally determined gender categories (Zuzelo, 2014).

For nurses to provide safe, ethical, compassionate and competent care, they must be aware of issues faced by people who are transgender (White Hughto et al., 2015). Many people living with gender diversity feel invisible, which can be mentally and emotionally painful. Of course, a person is much more than their gender; however, failing to acknowledge one's gender identity can create distress and contribute to gender‐related stigma, which can harm a transgender or queergender person's health and sense of well‐being (Rutherford et al., 2012; White Hughto et al., 2015).

Some would argue that by attracting a more gender‐diverse workforce, nursing can become more inclusive and reflect the population it serves (Smith et al., 2020). Recruitment, training, education and retention change would be needed to achieve this (Thompson et al., 2020). Normalizing conversations about the impact of gender, ethnicity, sexuality and other constructs would enable nursing students to learn to be conscious of the social processes that intersect to affect an individual's experience (Hankivsky, 2012; Zuzelo, 2014).

The history of nursing and sex/gender has tended to focus on the binary of female/male, which are sex‐based distinctions rather than gender‐based ones. The narrative is evolving as our collective understanding of gender expands. Moreover, the terminology of sex is often used interchangeably with gender and is often binary (Beckie et al., 2022). However, there is a shift to make a distinction between sex and gender terminology as this allows those identity components the uniqueness that they deserve and need to be better understood and incorporated into healthcare (Velasco, 2022). Not long ago, it was not unusual for someone who identified as “queer” to lose their job, be labelled as being mentally unwell or be imprisoned (Herek, 2007). Today's notion of duty of care within the nursing profession takes a different approach and continually strives to address and engage with the existing diversity.

## 3 THE STUDY

### 3.1 Objectives

- To explore university nursing students’ and academic staff's perceptions of what increased gender diversity might bring to the nursing profession.
- To better understand what increased gender diversity might bring to nursing and health care.
- To explore people's perceptions of why people of diverse genders do or do not apply for nurse education.
- To explore how the lack of gender diversity in nursing might impact health care delivery.

## 4 METHODS

In this study, an exploration was conducted among nursing students and academic staff within two UK university nursing schools, exploring their perceptions of what increased gender diversity might bring to the nursing profession. The COREQ was used as a guide throughout data collection and analysis (“COREQ (COnsolidated criteria for REporting Qualitative research) Checklist,” 2023). A survey and an interview approach were chosen for this study as the aim was to gain a better and deeper understanding of this issue and gather various viewpoints from those working and training in this field (Gerrish & Lathlean, 2015).

A working group (*n* = 8), nursing students (*n* = 2), service user (*n* = 1), nurse clinicians/academics (*n* = 4), and a researcher (*n* = 1) with diverse gender identities (women *n* = 2 she/her, men *n* = 3 he/him, non‐binary *n* = 2 they/them, transgender woman *n* = 1 they/them) was formed in the summer of 2022 to guide the study. The group met monthly throughout the study. Meetings allowed for gaining individual input on how the study should progress. Ideas were suggested, documentation was reviewed, survey and interview questions were developed, and findings were discussed. BQ (academic) led the research, and JM (researcher) conducted the interviews and analysed the data.

Data were collected from a relatively small population due to the time constraints of the study; however, it is hoped that the findings from this study will be used to provide an evidence base for continued research in this field. By using a small sample size, we could address the study question in a shorter time. We used this small sample size to gather results, which we hope to use to build upon in future research. The study had two stages:

- Stage 1: An online survey with nursing students and university nursing staff from two nursing schools (October–November 2022).
- Stage 2 Semi‐structured one‐to‐one interviews with students and university nursing staff in two schools of nursing (January–February 2023).

The survey and interview were used to help explore what people thought about gender diversity in nursing, health, and social care, as well as their colleagues' and respondents' personal experiences. Results were collated anonymously and used to gather concrete ideas and possible ways forward to help make the nursing profession more inclusive and what could be gained.

### 4.1 Stage 1: Online survey

An online survey was constructed using an iterative approach with input from the project team. Once ethical approval was received, the survey was uploaded onto Qualtrics XM (2023). The survey took ~10 min to complete. It included questions on what respondents understood about gender diversity, how gender‐diverse the respondents' colleagues/patients/students are, actions needed to increase gender diversity in nursing, barriers to attracting this diversity, opinions on education content concerning gender diversity, opinions of gender diversity in the nursing profession. At the end of the survey, there was an option to provide an e‐mail address should the respondent wish to participate in stage 2 of the study, which was an interview.

The survey was e‐mailed to all students and academic staff in the nursing schools of each university and was promoted by student and staff representatives. The recruitment e‐mail included a flyer which displayed a link to the survey as well as a QR code for ease of access. A participant information sheet (PIS) was attached to the e‐mail, which provided information on the purpose of the study and guidance on how to complete the survey. Appropriate contact details were included for those requesting further information. Further dissemination occurred by the project team; no mechanism was available to track this extended dissemination's reach.

The survey was open to all nursing students and academic staff as the topic of interest was focused on the experience of gender diversity in the nursing profession as well as barriers they had encountered to this. The survey was open from 16th November to 8th December 2022 and contained closed and open‐ended questions with the option to save and return later. Question 9 of the survey asked the respondent to provide their e‐mail address should they wish to be part of the next study stage: a one‐to‐one interview.

### 4.2 Stage 2: Semi‐structured interviews

An interview schedule was designed for this study based on the survey results, the aim and objectives, and input from the project team. Semi‐structured interviews were chosen for this study as they allowed the interviewer to converse with participants using their own words. They are most commonly associated with collecting qualitative social data when the researcher is interested in people's experiences (Bryman, 2012; Ritchie et al., 2014). An interview schedule was used to help the researcher by outlining what will be explored whilst allowing flexibility for details unique to each participant being interviewed (Marshall, 2016).

Nine survey respondents provided an e‐mail address to express their interest in being interviewed. However, only four consented to take part. Therefore, a further recruitment e‐mail was sent to staff and students, providing details of the study and contact details. Interviews occurred until the project team agreed that data saturation had been reached. The research team agreed upon data saturation when the same concepts were being discussed during the interviews, so further data collection seemed unnecessary. Ten individuals were interviewed. A pilot interview took place; however, no further changes were made to the schedule. Interview data collection took place between January and February 2023 on Microsoft Teams. Informed consent was obtained before each interview, which included permission to audio‐record the interview. The interview schedule provided a guiding framework to encourage ideas from the interviewed individual. Probes were used in response to the information provided by the individual. Microsoft Stream provided a draft transcript, which was edited and transcribed verbatim by the interviewing researcher as soon as possible to maximize the accuracy of the transcription process.

The data were analysed using thematic analysis (Braun & Clarke, 2006). Thematic analysis involves searching across a data set to find repeated patterns of meaning (Braun & Clarke, 2006). It is a flexible approach to analysis, making it a useful tool for providing a rich and detailed account of data (Bryman, 2012). The freedom this approach allows often attracts critique, as there are no clear and concise guidelines to follow. As thematic analysis is not linked to any pre‐existing theoretical framework, it can offer a more accessible form of analysis because it can be used with many theoretical frameworks (Braun & Clarke, 2006).

### 4.3 Ethics approval and consent to participate

The study received ethical approval from the universities on 11 November 2022. All respondents consented to participate in the survey by actively ticking consent boxes before they could proceed with the online survey. Before each interview, the participant was asked to read and sign a consent form.

## 5 FINDINGS

### 5.1 Survey

One hundred and forty individuals took part in the survey. Although complete responses were encouraged, respondents were advised to skip any questions they chose not to disclose. Thirteen per cent (*n* = 18) of respondents were staff, with most identifying as students (86%, *n* = 119). The remaining chose not to disclose this information. The survey was available for 3 weeks, from November to December 2022.

### 5.2 Characteristics of respondents/participants

Seventy‐one per cent (*n* = 99) of respondents identified as female, 24% (*n* = 34) as male, 2% (*n* = 3) as non‐binary, and 2% (*n* = 1) “preferred to describe themselves as” [*non‐binary trans‐femme, genderqueer*], 1% (*n* = 1) of respondents preferred not to say. Most respondents identified with the pronouns “she/her” (74%, *n* = 103), 23% (*n* = 32) he/him and 3% (*n* = 4) they/them. Over 70% (*n* = 98) who participated in the survey were aged between 18 and 34 years, and over 70% (*n* = 98) identified as “heterosexual/straight”.

Respondents were asked about their understanding of gender diversity [*How would you describe your understanding of “gender diversity” (women, men, trans and non‐binary persons?)*]. Ninety‐two per cent (*n* = 119) of those who answered said they either “felt confident” or “understood the main concept of gender diversity”. When asked about education related to gender diversity, only 33% (*n* = 33) of respondents said that their education in the clinical environment, either as a nurse, that is, a student or teaching faculty member, included content on “gender diversity”. The number of students and staff reporting exposure to education within the university school was slightly higher at 46% (*n* = 46).

Only nine per cent (*n* = 12) of respondents described their team of colleagues as being “extremely gender diverse” whilst 13% (*n* = 17) stated that there was “no gender diversity apparent” in their team. The majority indicated a response between these two extremes. Respondents were also asked about gender diversity among those they cared for: 16% (*n* = 16) of respondents stated that there was no gender diversity among those they cared for, whilst 18% (*n* = 19) reported that the people they cared for were “extremely gender diverse”. However, most respondents (63%, *n* = 66) did not view the patients they care for as “gender diverse”.

Mindful that only 13% of respondents identified as staff, 19% of respondents stated that there was little or no gender diversity apparent in the student body they taught. In comparison, 16% (*n* = 6) of respondents stated that the students they taught were extremely gender‐diverse.

Sixty‐four per cent (*n* = 69) of respondents strongly agreed/agreed that the lack of gender diversity in nursing negatively impacted delivering care to a gender‐diverse society. In contrast, only 16% (*n* = 18) of respondents reported that they did not think the lack of gender diversity affected nursing care delivery. Eighty‐four per cent (*n* = 90) stated that they strongly agreed/agreed that increased gender diversity within the nursing workforce would be a positive attribute of nursing. At the same time, 8% (*n* = 8) strongly disagreed/disagreed with this statement. However, 56% (*n* = 55) strongly agreed/agreed that the current nursing workforce could care for patients with diverse gender identities. Forty‐three per cent (*n* = 42) strongly agreed/agreed that their gender impacted the nursing care they provide, whilst 35% (*n* = 35) of respondents strongly disagreed/disagreed with this statement. The majority of respondents, 75% (*n* = 74), strongly agreed/agreed that working with a more gender‐diverse nursing workforce would enrich their experience as a nurse.

A full profile of the demographics collected can be found in Table 1.

**TABLE 1:** Demographics of survey respondents.

	*N*	%
Gender		
Male	34	24.29
Female	99	70.71
Non‐binary	3	2.14
Prefer not to say	1	0.71
Prefer to describe myself as:	3	2.14
Pronouns		
He/him	32	23.02
She/her	103	74.10
They/them	4	2.88
Age		
18–24	49	35.25
25–34	50	35.97
35–44	17	12.23
45–54	13	9.35
55+	10	7.19

The survey respondents were asked about barriers that might prohibit greater gender diversity in nursing. The majority of respondents felt some barriers needed to be addressed, for example, stigma and stereotyping, “*Stereotype of nursing profession as a female profession due to the start of it*”. However, a small minority felt there were no barriers. One respondent stated, “I don't think there are any barriers; *it is an open profession provided you meet the appropriate criteria and pass the relevant interview/application process. Your gender should not influence this; we become nurses to provide care and follow our code regardless of gender*”.

Survey respondents were invited to suggest concrete actions to promote and attract a more ‘gender diverse’ nursing profession. Many suggestions were made including, *‘More awareness, being taught what it is, as some people may not have an understanding’*. However, a few respondents did not think action was required. One respondent stated, “*I don't think we should aim at this beyond attracting more men into nursing. People who consider themselves gender diverse should be able to enter nursing, but I don't think the ideology attached to gender diversity is good for individuals or society. It should not, therefore, be imposed on patients and colleagues*”.

After analysing the survey findings, the project team met to discuss them and agree on some guiding questions informed by the findings to guide the interview process in stage 2 of the project. Microsoft Word generated tables and charts to add demographic and statistical information to this study stage.

### 5.3 Interview results

Each participant's identity was protected using a coding system, participant P1–P10. A process of thematic analysis (Braun & Clarke, 2006) took place. Each recording was transcribed using Microsoft Stream, and the transcripts were read and edited to provide an accurate account of the discussions. Each transcript was then read and reread to get an overall view of emerging themes. The data were coded, and themes were formed, which were discussed by the two team members with access to the raw data. The study leads and lead researcher conducted quality checks before presenting the initial themes to the project team. Only two project team members (lead researcher and study lead) saw the raw data to ensure the confidentiality of those who took part in the interview, as agreed by the research protocol and the ethics panel. The project team discussed the initial emerging themes with some participant quotes, and four key themes emerged.

#### 5.3.1 Theme 1: Shared stereotypes

Most of those interviewed spoke about their perception that nursing has always been largely a profession of women. It was seen as women's work and was associated with stereotypes. Participants believed that this perception also influenced how patients saw the nursing profession.

A final year nursing student (P10, he/him), who identified as male, said he understood that nursing was considered largely a woman's profession when he embarked on his studies. The participant said his 5‐year‐old niece was surprised to hear he was training to be a nurse. At this young age, his niece associated nursing with women. Another participant (nursing student, she/her) highlighted that within school, career choices are very gendered, “…*I studied at a grammar school, and it was very much like the girls did nursing and the boys did medicine and like, I'm only in my mid‐20s and that was the perception*” (P4, she/her). Both participants clearly stated that children from an early age needed support to see that nursing was a job option for everyone, regardless of gender.

Participants agreed that nursing is viewed as a woman's profession based on a historical and traditional view, which has become ingrained in public knowledge over time.

Lots of stereotypes that exist around gender and gender roles, and there's a lot of people that still, you know, reminisce on the past, like the good old days like. [P8, nursing student they/them]

Another respondent agreed that, “*… society still deals in stereotypes*” (P2, staff, he/him) whilst another respondent (P8, nursing student, they/them) stated that patients often mistook nurses who are men for doctors, “*Patients automatically think that they're doctors, because they're men*”.

Most of those interviewed thought that a patient's response to being cared for by a nurse of a diverse gender varied greatly from one person to another. Most participants believed that the majority of patients would be more focused on and concerned about the standard of care they received. There was, however, a recognition that some patients would rather be cared for by someone with a gender they perceived to be similar to themselves, as the nurse might be better able to relate to them.

Yeah, I think it's probably difficult. I think generally people would want someone like them to treat them because that's when they would feel the most comfortable. [P1, nursing student, they/them]

Another participant (P8, nursing student, they/them) commented that some patients have a “*closed‐minded view*” and still have prejudices about those with diverse gender identities. However, P6 [staff, he/him] stated that they thought patients' views would vary greatly and that some would be “*absolutely delighted*” to see those with diverse gender identities joining the nursing profession.

P10 [nursing student, he/him] explained that his experience has been that some male patients are relieved to see a nurse who is a man on the ward as they feel uncomfortable around so many staff who are women. He continued that it is generally accepted that nurses who are women can provide intimate care to any gender of the patient. Still, nurses who are men tend to feel they must check that the patient is comfortable with the situation first. The same participant felt that many of his female nursing colleagues might have assumed that all patients were comfortable with them delivering intimate personal care. In contrast, he was mindful of checking with the patient to see if they were comfortable with him carrying out these intimate aspects of care.

P1 [nursing student, they/them] highlighted that in care homes, patients are often segregated by gender (men/women), and policies are in place where men can provide personal care to women and women can provide personal care to men. However, the participant felt that trans and non‐binary people were treated differently as nobody wanted personal care from them at all.

…It just seems like there is this over sexualization of gender differences. It was like because you're trans, you must be hypersexual. Therefore, you can't provide personal care to someone, whereas men are also hypersexual and can't provide personal care to women…. [P1, nursing student, they/them]

Participants recognized that nurses' perceptions of gender diversity might differ between generations. Some participants thought that older generations of nurses might struggle more with gender diversity than younger people. However, P10 [nursing student, he/him] has experienced a mix of views between generations and felt that nursing staff who have grown up with particular views should be challenged, as being brought up to believe something does not mean it is right.

There was some consensus among participants that as the current nursing education cohort has been exposed to ‘PRIDE celebrations’, ‘homosexuality’ and ‘gender diversity’ growing up, they tend to be more open‐minded and that greater acceptance exists among this generation of nurses and nursing students.

Because our generation is, you know, we've brought up with this sort of movement and PRIDE and everything where we bit more open to other ideas and like acceptance. [P5, nursing student, she/her]

Conversely, some older nurses who were interviewed and had been in the profession for some time recognized that many of these concepts were new to them, so they could, at times, respond with resistance.

P3 [*staff, she/her*] said much has changed in the last 10 years and that for colleagues who have been working in nursing for a long time, this could be a difficult shift and change in thoughts as perceptions become ingrained over time.

The idea of trans was just never there, you know, LGBTQ was literally it. There was no place. There was nothing additional. So even within 10 years, how much has changed… [P3, staff, she/her]

Interestingly, although the participant felt that ‘the idea of trans was just never there’, the ‘T' in the term ‘LGBTQ’ she used, which stands for transgender, was not recognized. A few participants suggested that the media has a role in how gender diversity is perceived and how nursing is portrayed as a career choice. P8 (nursing student, they/them) suggested that the media representation of nurses needs to be improved.

P6 [staff, he/him] said nursing needs to reflect what is going on in society, and hopefully, this will help to bring about change gently. P2 (staff, he/him) suggested that as society changes, nursing should “change with it.” However, nursing should not rush to change society, as it is not the profession's responsibility to use vulnerable people to change views.

I don't know that nursing should rush at it, as if, as if we are sort of at the forefront of changing society through the medium of the experience of vulnerable people. [P2, staff, he/him]

#### 5.3.2 Theme 2: Improved care through knowledge, better relations and the presence of a gender‐diverse workforce

Throughout the interviews, it became apparent that many participants thought that the gender of the nurse and the gender of the patient had an important role in delivering person‐centred care in nursing. Some participants saw the life experience of the nurses as an important component of delivering person‐focused care, which was seen as very positive.

…we all bring our own life experiences or the code like cultural and contextual things that we've lived through into nursing. And, you know, being a good nurse is being able to bring that in and it's effective, but also not coming from it from a like a personal point of view. [P3, staff, she/her]

Therefore, it was perhaps not surprising that most participants recognized this important link and that gender diversity must also be considered to provide true person‐centred care. “*Look at that person as a whole and all of the parts that make up that person and make them who they are…*” (P8 nursing student, they/them).

As part of person‐centred care, P10 [nursing student, he/him] believed that many patients appreciate the opportunity to sit down and talk about themselves to give them the care they deserve. They need to be understood, including their gender identity, and treated as individuals. Just as patients wanted to be understood, spending time with them was also seen as an opportunity to learn about gender identities.

…if, you know, you were working in a certain area and you had a trans person who come in, you know, that's a prime learning opportunity. You know, you sit with that person, you explore what's important to them. [P10 nursing student, he/him]

Another participant thought that person‐centred care could be improved with more gender diversity within nursing teams.

…I think you could get better person‐centred care maybe for you know LGBTQ Plus communities if we've got a wider range of nurses coming in. [P9, nursing student, he/him]

However, one participant [P5, nursing student, she/her] expressed concern that person‐centred care was ‘*close to non‐existent*’ due to lack of time. Another participant (P4, nursing student, she/her) agreed that it was difficult to provide this level of care due to the health service crisis, including a lack of resources.

you always leave feeling like you haven't fully given person‐centred care because you might not have been able to sit with that person for longer than 5 minutes…but you're sitting there and you're trying to give them their time and your efforts of trying to console them but in the back of your head, you're like, don't sit here too long because you can't, you know. (nursing student, she/her)

There was general agreement among most participants that many benefits could result from an increase in gender diversity within the nursing profession. P4 (nursing student, she/her) believed that a diverse gender profession with a gender‐diverse society could only lead to better things, although they did not specifically mention what these good things would be.

When referring to gender diversity, some of those interviewed saw this as simply men and women. One of these, P2 (staff, he/him), focused on men and women only; they suggested that having men in nursing adds a compliment to the profession and that these two genders work well together, both bringing their own unique skill set, “*…women bring something to healthcare that men don't, and men bring something that women don't*”. P2 (staff, he/him) went on to state that we cannot fully know what an increase in gender diversity would bring to nursing. Still, we can speculate that “*…if more men went into nursing, it might raise the status of nursing because typically men have a higher status in society. You know, that would be one argument*”.

However, P1 [nursing student, they/them] appeared to refer to the broader gender diversity that exists when they spoke about how the mix of gender within nursing brought a greater strength to the profession:

…the more diversity we have within nursing as profession, the broader scope of skill we develop in terms of interpersonal relationships and relating to and with different people. And I think it's super important that we're able to have colleagues within our ranks that are of all different experiences.

Another participant suggested that groups of men could often behave in a *‘macho’* manner whilst women could be *‘bitchy’*. However, bringing more diverse gender groups, beyond the notion of male and female, to nursing could help to dilute these unhealthy situations and bring more balance to the profession, “*…we need them really good, experienced females or non‐binary or whatever people to come in to dilute that*”.

Some participants suggested a better representation of the general population entering the nursing profession would benefit patient care. Not everyone fits the stereotypical view of what a nurse is, and it is important that patients with diverse needs feel they and their gender are understood by those caring for them and that the staff can approach their problems.

…they feel like the nurses caring for them don't really understand. You know, they don't know how to approach their problems. They don't know how to think about, you know, their psychology and the way they might be thinking and feeling about things. [P10, nursing student, he/him]

Another participant also saw this idea of a more gender‐diverse nursing workforce better understanding patients' needs as very positive. She suggested increasing gender diversity in nursing would positively affect relationships, as those with diverse gender identities would feel more listened to and accepted.

I definitely think that it would help you know patients like service users who identify as trans and non‐binary. I definitely feel it would help them come forward, feel more comfortable, feel more accepted and know that they're listened to and like taken seriously. [P5, nursing student, she/her]

Although some participants had different ideas of what gender diversity might mean in nursing, there was a consensus that nursing would be much poorer without an increase in gender diversity. Suggestions were made that nursing could alienate groups of people if the nursing profession did not reflect these gender groupings.

….we sort of risk alienating groups of people that we do look after and then for me it's sort of hand in hand with then nursing not really serving the populations that we're meant to serve. [P3, staff, she/her]

I think what can be missed as that there are people that are living currently in society that don't feel like they belong anywhere. [P4, nursing student, she/her]

It was suggested that if patients of more diverse genders do not experience nurses who can relate to them, they may feel they do not belong and therefore may be less willing to trust the nurse and seek help when needed.

… if you don't feel like you belong anywhere, it's very difficult, obviously, to open up and, you know, seek help… (P4, nursing student, she/her)

#### 5.3.3 Theme 3: A culture of welcome: Suggested changes for the future

Most participants recognized the richness that could come from greater gender diversity within nursing, reflecting the gender‐diverse society the profession services. Most participants agreed that more could be done to make the nursing profession a more welcoming and inclusive career for those of diverse genders. There was a sense among participants that small steps, in time, would lead to a positive change in nursing in terms of gender diversity.

You keep sowing seeds for change … people are so much more comfortable and open talking about things than 20 years ago… [P6, staff, he/him]

Some small steps included more gender‐diverse representation at recruitment, more flexibility around admission procedures and ward allocation, gender‐neutral toilets, gender‐neutral uniforms, the use of pronouns and changes in language use.

It was suggested that recruitment fairs and promoting nursing as a career choice should involve input and visual representation of those from diverse gender groups. More openness and hearing from those already working as a nurse could create a more welcoming environment and encourage others to join.

If there was a nurse … they could like reassure and say, look, I identify as trans or non‐binary and I work in this setting and this is how it is. [P5, nursing student, she/her]

It was proposed that images used in recruitment strategies should be sensitive to gender diversity and display the level of diversity that nursing strives for. It was proposed that awareness of individuals' preconceived ideas and biases should be considered when planning recruitment events to ensure all genders realize they could have a place in nursing.

Several participants discussed admissions and ward allocation in relation to healthcare. Not all admissions forms provide a place for patients to stipulate their pronouns; the current format is rigid and would benefit from more flexibility.

I think it's still very rigid and that you're a male or a female, but it shouldn't be like that. [P5, nursing student, she/her]

Participants spoke of folders assigned to patients on admission as blue for men and pink for women. This simple stereotype presents another difficulty for those patients identifying with diverse genders, leading to exclusion. Many expressed their concern about how this might make a patient feel.

There was much agreement that nurses' uniforms should be gender‐neutral, and many spoke of the welcome move to scrubs during the pandemic. Having two uniforms, one for men and one for women, seemed pointless and exclusive to most participants. For some, it created a real problem, particularly for non‐binary staff members who faced a predicament of which uniform to choose.

I think that separate uniforms are pointless. Gender‐neutral ones would be better. [P8, nursing student, they/them]

Conversations about the need for gender‐neutral toilets arose frequently. Staff and students are often forced to choose between a male and female toilet. However, P7 (nursing student, he/him) said the toilets are starting to change to unisex and that this “*shows a level of maturity and that we don't need to fight about toilets*”.

Views were voiced that education on gender diversity should be mandatory for all those entering the nursing profession and that there should not be a reliance on those in minority groups to educate others.

Stop making them in that sense have to define what their identity is whenever we don't expect anybody else to. [P10, nursing student, he/him]

Improved training for staff would be welcomed so they become better educated in EDI and a zero‐tolerance policy for gender discrimination would be implemented.

Several participants mentioned the helpful use of pronouns and could see the importance of being more inclusive and changing attitudes in general. P10 [nursing student, he/him] said it is about finding the “*appropriate language*” and ensuring we become more comfortable using it. Making a conscious effort to use the correct pronouns and apologize when you get it wrong.

… if you get it wrong, you know, apologise for it and you know try and move on from it there because otherwise you know you're not actually doing what you're doing a lot of injustice that was and sense to that person who's told you something particularly important to them. [P10 nursing student, he/him]

P8 (nursing student, they/them), a non‐binary nursing student said they did not feel comfortable enough to display their pronouns at work and would rather not have those conversations in the workplace and would not correct anyone over pronoun use.

I don't correct anyone at work. I don't wear them [pronouns] … It opens up conversations that I'd rather not have…. [P8 nursing student, they/them]

## 6 DISCUSSION

The participants in the study were nursing students and academic staff who had the experience of learning in a university setting and providing nursing care in clinical practice. Although most people who took part in the study described their gender as male (*n* = 34) or female (*n* = 99), a few people (*n* = 3) identified their gender as non‐binary. This finding is unsurprising as nursing continues to be a profession chosen predominantly by people who identify as a woman, and the gender diversity present in society is absent (Eliason et al., 2010). As the conversation around gender diversity continues to grow and be heard within nursing, health and social care, and in society, more people may feel greater freedom and feel safer to explore, examine and express their gender identity and perhaps to become part of the nursing profession.

The majority of respondents reported in the survey that they understood the concept of gender diversity and recognized the richness of gender diversity within society in health care and nursing and the benefits this could bring. This contrasts with the “nursing silence” reported in a study just over 10 years ago, when nurses were reluctant to engage in similar conversations (Eliason et al., 2010). However, more than 10 years later, during the interviews, some participants appeared to speak about the importance of gender diversity, but with a binary focus on women and men. This might suggest and support earlier research that more should be done around raising awareness in nursing and that more work should be done on creating greater awareness of what gender diversity really means. It may also reflect the lack of training about gender diversity within the university and clinical setting referred to in the findings.

Some of the participants in the interview did indeed recognize that gender diversity was much broader than the concept of men and women and addressed the presence and beauty of gender diversity, including transgender, queergender and non‐binary persons. These beliefs are similar to the thoughts of medical and nursing students and staff, who took part in an earlier workshop exploring the needs of transgender persons in health care (Corrigan et al., 2020). In that work, those who attended the training voiced their commitment to making health and social care a more inclusive and welcoming space. It should be recognized that many students and staff chose not to engage in this earlier training, just as many chose not to participate in this study. These voices still need to be heard, their views listened to, and ongoing dialogue is required.

The study findings also suggest that the perceptions of gender diversity within the nursing profession might be influenced by the society, the culture, the generation, and the training each person is exposed to. This cultural and societal influence might also explain why many lesbian, gay, bisexual and transgender (LGBT) people believe that health and social care services cannot respond to their needs (Bachman & Gooch, 2018). There is a real danger if this is the perception held by many LGBT persons that they may choose not to access services where they do not feel welcome or understood or may choose not to disclose intimate information that affects their health and well‐being, fearing the health care professionals' response. This may also be true of people whose gender is not always recognized by society or by nursing teams and healthcare services.

Most participants in the study believed that gender diversity was missing from nursing, and some university nursing staff believed this was missing from the student body they taught. Gender diversity might be absent, but as one participant indicated, people may not feel comfortable revealing their gender identity in either the university or clinical setting. This spoken fear reflects the findings from previous work when young people were frightened to reveal their true gender identity (McDowell, 2021; Merryfeather, 2014). Most people (84%) in this current study believed that nursing and health and social care would be much richer if the profession better reflected the gender diversity of those that nursing serves. These findings support the thoughts of Quinn et al. (2022) in recognizing that the lack of gender diversity within the nursing profession must be addressed (Quinn et al., 2022). Some participants in the current study felt less comfortable with a notion of gender diversity that went beyond that of a woman or man, and their thoughts and ideas may reflect many voices within society today. Whilst these voices need to be heard and respected, some participants in the interview believed that part of their role was to challenge colleagues to move beyond this narrow concept of gender diversity and to embrace the richness that exists within society and those the profession cares for. If the profession does not address this issue, people accessing health services may continue to feel excluded and not seen (Bauer et al., 2014).

There was a recognition in both the survey and interviews that many barriers continue to exist, barriers that exclude people from being part of the nursing profession. This might include the lack of visibility of gender diversity, the use of binary language or having to choose a uniform that only recognizes men and women. Similar barriers might also mean patients' needs are not always addressed; the United Kingdom Stonewall survey (2018) findings support this. Some participants in this current study believed that the more the nursing profession was visibly gender diverse, the more willing people from diverse experiences who require nursing care might feel safe to acknowledge their hidden needs. This might enable those who do not have confidence in healthcare services to share their concerns (Bachman & Gooch, 2018).

The concept of moving from a task‐focused healthcare system to one that focuses on the individual has been present for many years (McCormack, 2020). However, the important link between person‐centred care and gender diversity has not always been focused on within nursing. In this study, it was an important focus within the findings. Although some participants felt that delivering true person‐centred care continued to be a challenge due to current demands, an important part of person‐centred care was recognizing and respecting each person's gender identity, the gender of the patient and the gender of the nurse. It was also recognized that each person was much more than their gender identity and should not be labelled by this. However, each person's gender identity is an intrinsic part of their identity. One does not leave one's gender behind when performing the role of a nurse or when receiving care as a patient. Perhaps, some of the taken‐for‐granted importance of one's gender identity only becomes apparent when one is forced to conform to gender stereotypes. This might include making difficult decisions about conforming to a dress code that does not recognize one's identity or feeling excluded by a caring profession one wants to be a part of. Or having to confront the ongoing binary segregation of women and men, which is a part of society and reflected in many university and hospital settings, including the choice of gender‐segregated toilets and wards.

An important finding in this study is that no person, whether they identify as woman, non‐binary, trans or man should presume that a patient is comfortable with that nurse looking after their personal and intimate needs. Suggesting that all nursing staff require greater sensitivity and awareness, including seeking permission before carrying out intimate care needs, just as any other clinical task.

The study suggests that in this group of nursing students and academic staff from two universities, there was an openness and a willingness to learn and to do better. The visibility of gender diversity, whether at recruitment drives, advertisement campaigns, health and social care settings and in the language used, must be much more inclusive. This means moving beyond tokenism to reality. This means ensuring that all universities that provide nursing programmes are engaged in this important conversation. This might begin with inviting each person within the nursing profession to explore the beauty of their gender and how it influences who they are and how they nurse. It might also mean reflecting on those whose gender, a core part of who they are, has not been recognized by society, leading the person to feel excluded, ostracized and hurt.

Every person interested in joining the nursing profession should see clear signs of welcome, diversity and inclusion at every step of the recruitment process. This means no longer stereotyping the nursing profession by outdated images. A profession built on the core concept of care (Quinn, 2017) and a profession that reflects the gender diversity of the people it serves. This visibility will attract more people into the profession, and those who need nursing care will feel better understood and be more willing to trust those caring for them with their needs.

## 7 LIMITATIONS

The data collection procedures included surveys and semi‐structured interviews, which were promoted online via the two university schools of nursing; other dissemination methods may have helped to generate more respondents/interviewees. The time constraints of the allocated funding for this project meant the survey could only be opened for a short period, limiting the responses received. To be eligible for the interview, individuals had to be English speakers; this may have introduced bias to the sample in that potential interviewees may have been excluded, but due to the language skills of the research team, this was an essential criterion.

## 8 CONCLUSIONS

New insights as to what is known about gender diversity in nursing and the need for positive changes have been highlighted. As echoed in previous research, this study recognizes the importance of listening to all voices within the nursing community. The survey and interviews have provided insight into the views held by students and staff from two nursing schools and have identified that increased gender diversity would be a positive step forward for the nursing profession.

There appear to be ongoing barriers to making nursing a more gender‐diverse profession. Therefore, clear areas to target would be the advertising and recruitment process, staff training and reducing stigma. Including those with more diverse genders within marketing strategies and recruitment fairs would help people view nursing as a career choice suitable for all. Education for staff would focus on developing understanding, raising awareness, and introducing gender‐diverse language, which would be a welcome addition to the profession. Furthermore, a zero‐tolerance policy for gender discrimination should be implemented. Practical issues such as staff uniforms and the rigid systems often associated with nursing should be reviewed and transformed to become more inclusive, reducing stigma for staff and patients. A more gender‐diverse nursing workforce would help create an environment where patients are better understood and true person‐centred care could be implemented.

This research could be a useful platform for further complementary studies with a wider population, including students, nurses, service users and family members.

## AUTHOR CONTRIBUTIONS

Made substantial contributions to conception and design, or acquisition of data, or analysis and interpretation of data: J.M., D.R.T., A.D., A.P., T.D., P.J., C.M., B.Q. Involved in drafting the manuscript or revising it critically for important intellectual content: J.M., D.R.T., A.D., A.P., T.D., P.J., C.M., B.Q. Given final approval of the version to be published. Each author should have participated sufficiently in the work to take public responsibility for appropriate portions of the content: J.M., D.R.T., A.D., A.P., T.D., P.J., C.M., B.Q. Agreed to be accountable for all aspects of the work in ensuring that questions related to the accuracy or integrity of any part of the work are appropriately investigated and resolved: J.M., D.R.T., A.D., A.P., T.D., P.J., C.M., B.Q.

## FUNDING INFORMATION

This study was funded by The Burdett Trust.

## CONFLICT OF INTEREST STATEMENT

The authors declare that they have no conflict of interest.

### PEER REVIEW

The peer review history for this article is available at https://www.webofscience.com/api/gateway/wos/peer‐review/10.1111/jan.16246.

## ETHICS STATEMENT

This study received ethical approval from the universities in which the study took place.

## Data Availability

The datasets generated and/or analysed during the current study are not publicly available due as they contain potentially identifable information but are available from the corresponding author on reasonable request.
